# Long-term outcomes according to absolute value vs. percentage reduction in low-density lipoprotein cholesterol levels after acute myocardial infarction

**DOI:** 10.3389/fcvm.2025.1653447

**Published:** 2025-12-05

**Authors:** Kyung Hoon Cho, Jung Ho Yang, Sang Yeub Lee, Min-Ho Shin, Seok Oh, Min Chul Kim, Doo Sun Sim, Young Joon Hong, Ju Han Kim, Youngkeun Ahn, Jang Hoon Lee, Ju-Seung Kwun, Young-Hoon Jeong, Joo-Yong Hahn, Jin Yong Hwang, Myung Ho Jeong, Weon Kim

**Affiliations:** 1 Department of Cardiology, Chonnam National University Hospital, Gwangju, Republic of Korea; 2 Department of Cardiology, Chonnam National University Medical School, Gwangju, Republic of Korea; 3 Department of Preventive Medicine, Chonnam National University Medical School, Hwasun, Republic of Korea; 4 Division of Cardiology, Chung-Ang University Gwangmyeong Hospital and Department of Internal Medicine, Chung-Ang University College of Medicine, Gwangmyeong, Republic of Korea; 5 Department of Cardiology, Kyungpook National University Hospital, Daegu, Republic of Korea; 6 Department of Internal Medicine, School of Medicine, Kyungpook National University, Daegu, Republic of Korea; 7 Department of Cardiology, Seoul National University Medical School, Seoul, Republic of Korea; 8 Department of Cardiology, Sungkyunkwan University School of Medicine, Seoul, Republic of Korea; 9 Department of Cardiology, School of Medicine, Gyeongsang National University, Jinju, Republic of Korea; 10 Department of Cardiology, Gwangju Veterans Hospital, Gwangju, Republic of Korea; 11 Division of Cardiology, Department of Internal Medicine, Kyung Hee University Hospital, Kyung Hee University, Seoul, Republic of Korea

**Keywords:** LDL cholesterol, acute coronary syndrome, myocardial infarction, incidence, registries

## Abstract

**Backgrounds/aims:**

Real-world data are limited regarding long-term outcomes in terms of absolute follow-up values of low-density lipoprotein cholesterol (LDL-C) vs. percentage reductions from baseline after LDL-C-lowering therapy for patients with acute coronary syndrome. We aimed to investigate the associations between 5-year clinical outcomes and absolute follow-up LDL-C levels or percentage reductions from baseline using a nationwide Korea Acute Myocardial Infarction Registry (KAMIR).

**Methods:**

Of 13,662 patients from the KAMIR–National Institutes of Health database, we identified 6,248 patients who had documented LDL-C levels at baseline and during 18 months of follow-up. The primary outcome was major adverse cardiovascular events (MACE; a composite of nonfatal stroke, nonfatal myocardial infarction, repeat revascularization, and all-cause death) at 5 years.

**Results:**

In the analysis of absolute follow-up time-weighted average LDL-C levels (<55, 55–69, 70–89, and ≥90 mg/dL), there was a U-shaped trend of MACE incidence (10.8% vs. 9.3% vs. 10.0% vs. 13.2%, *P* = 0.003). In the analysis of the percentage LDL-C reduction from baseline, greater reductions were associated with lower MACE risk. In a multivariable Cox time-to-event analysis with LDL-C < 50% reduction from baseline as the reference, ≥50% LDL-C reduction from baseline was independently associated with a decreased incidence of MACE (adjusted hazard ratio, 0.76; 95% confidence interval, 0.62–0.92).

**Conclusion:**

This study involving 6,248 AMI patients demonstrated that the greater the LDL-C reduction from baseline, the lower the risk of MACE. However, there was no clear decreasing trend in the risk of MACE when absolute follow-up LDL-C levels were lowered from around 70 mg/dL

## 1 Introduction

Low-density lipoprotein cholesterol (LDL-C) lowering therapy has proved effective in the primary and secondary prevention of atherosclerotic cardiovascular disease (1, 2). Previous dyslipidemia guidelines in 2011 set LDL-C targets of “<70 mg/dL and/or ≥50% LDL-C reduction from baseline” or “<70 mg/dL” for patients with acute coronary syndrome (3, 4). Recent guidelines recommend LDL-C targets of “<70 mg/dL or <55 mg/dL” and “≥50% reduction from baseline” in this population (5, 6). However, global studies of LDL-C target achievement in real-world settings have used absolute follow-up LDL-C targets, such as <70 or <55 mg/dL, which have shown poor LDL-C target achievement rates (7–12). A recent survey in Korea regarding the optimal LDL-C targets for acute myocardial infarction (AMI) patients revealed that a considerable proportion of physicians prefer to use only absolute follow-up LDL-C targets of “<70 mg/dL or <55 mg/dL” without considering percentage LDL-C reduction from baseline (13). Furthermore, a recent 2023 European guideline emphasizes an absolute follow-up LDL-C level of 55 mg/dL to guide decisions about changing lipid-lowering therapy (14). However, data comparing LDL-C targets between percent reduction and absolute levels during the follow-up of patients with acute coronary syndrome are scarce. Therefore, we aimed to investigate the associations between clinical outcomes and percentage LDL-C reductions from baseline or absolute follow-up levels in the monitoring of LDL-C lowering therapy after AMI using a nationwide Korean registry with 5 years of follow-up.

## 2 Methods

### 2.1 Data sources and participants

**Figure 1:**
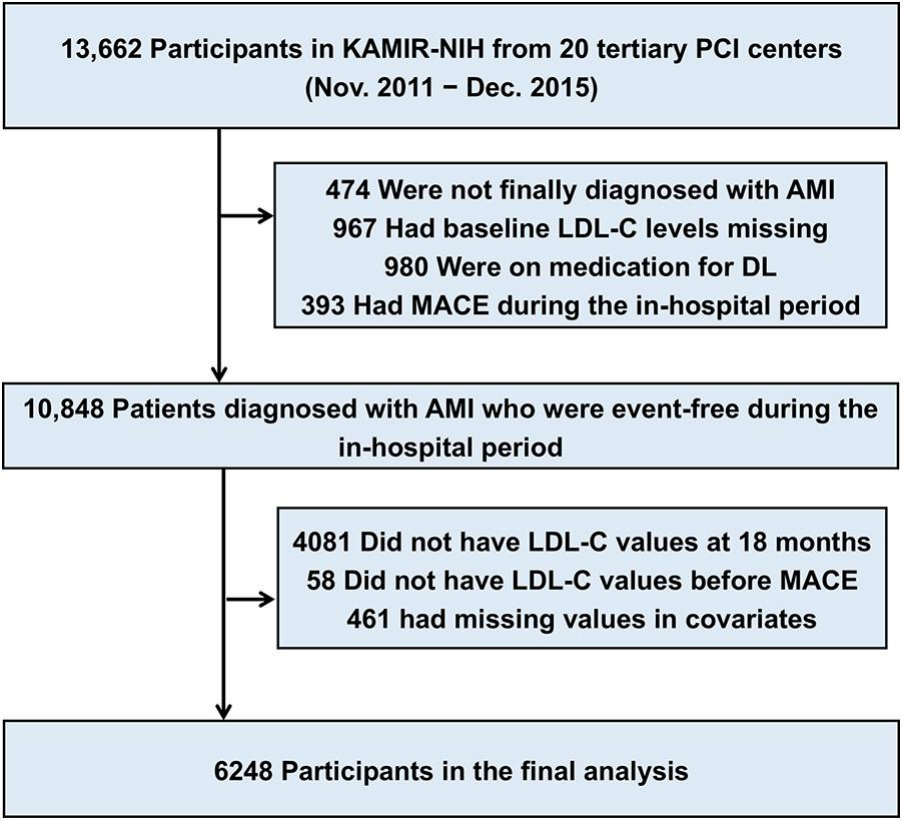
Description of the study population. The study cohort was derived from the nationwide prospective KAMIR (Korea Acute Myocardial Infarction Registry)-NIH (National Institutes of Health). AMI, acute myocardial infarction; DL, dyslipidemia; LDL-C, low-density lipoprotein cholesterol; MACE, major adverse cardiovascular event; MI, myocardial infarction; PCI, percutaneous coronary intervention.

The study population was derived from the Korea Acute Myocardial Infarction Registry (KAMIR)-National Institutes of Health (NIH), a nationwide prospective multicenter registry with 3 years of clinical follow-up that consecutively enrolled patients from 20 tertiary university hospitals in Korea between November 2011 and December 2015. Participating centers had facilities for percutaneous coronary intervention and onsite cardiac surgery (Supplementary Methods). The KAMIR-NIH-LIPID study was funded by the National Evidence-based Healthcare Collaborating Agency and was designed to reinforce the KAMIR-NIH data for investigating real-world practice on lipid-lowering therapy and 5-year clinical outcomes in patients with AMI. Between May 2022 and August 2023, clinical research coordinators retrospectively collected additional data on the electronic medical records of each participating center using an Internet-based Clinical Research and Trial management system (iCReaT), which was a data management system set up by the Centers for Disease Control and Prevention, Ministry of Health and Welfare, Republic of Korea (iCReaT study no. C110016). Of the 13,662 patients from the KAMIR-NIH, we identified 6,248 patients who were not on lipid-lowering therapy before hospital arrival and had LDL-C levels at baseline and at least once during 18 months of follow-up (Figure 1).

### 2.2 Outcomes and definitions

The co-primary outcomes were major adverse cardiovascular events (MACE; a composite of nonfatal stroke, nonfatal myocardial infarction, repeat revascularization by coronary artery bypass grafting, and all-cause death) and all-cause death at 5 years. Cardiac death was defined as any death not definitively non-cardiac. AMI was diagnosed based on the universal definition of AMI. ST-elevation myocardial infarction was diagnosed when there was a new ST-segment elevation of ≥0.1 mV in ≥2 contiguous leads (≥0.2 mV in V2–V3) or a new left bundle branch block with a concomitant increase in cardiac enzyme levels (troponin I/T or creatine kinase myocardial band). Statin intensity was defined according to the 2018 American guidelines on cholesterol management. Lipid profiles were measured directly as routine analyses at each center. We calculated the time-averaged LDL-C as the cumulative LDL-C divided by the total duration between admission and 18 months (15). It was assumed that LDL-C levels during specific periods were the levels available just after those periods. LDL-C levels were included only when they were recorded before clinical events occurred. Based on this result, follow-up LDL-C levels were classified into four strata: <55 mg/dL, 55–69 mg/dL, 70–89 mg/dL, and ≥90 mg/dL. Percentage LDL-C reductions from baseline were classified into three strata: no reduction or increase, 0%–50% reduction, and ≥50% reduction.

### 2.3 Statistical analysis

Categorical variables are described as a number of cases and percentages and were compared using the chi-square test or Fisher's exact test, as appropriate. Continuous variables are presented as mean ± standard deviation and were compared using a one-way analysis of variance or the Kruskal–Wallis test, as appropriate. Kaplan–Meier curve analysis of mortality and MACE was performed using a log-rank test. Multivariable Cox regression analysis was performed to address the association between absolute levels or percentage reduction of LDL-C and outcomes using an enter method, including the baseline variables judged to be of clinical relevance–specifically, age, sex, body mass index, hypertension, diabetes mellitus, previous myocardial infarction, previous revascularization, family history of premature coronary artery disease, smoking status, type of MI, and statin intensity. A Cox proportional hazards regression model using a restricted cubic spline function was used to assess the association between follow-up levels or percentage reduction rate of LDL-C and clinical outcomes in an adjusted model. Patients with missing values in covariates were excluded from the study flow. Furthermore, we examined the analyses stratified by sex. A two-sided *P* value <0.05 was considered to indicate statistical significance, and analyses were performed using Stata 16 (StataCorp, College Station, TX, USA) and R version 4.2.2 (R Development Core Team, Vienna, Austria). This study was reported in accordance with the STROBE (STrengthening the Reporting of OBservational studies in Epidemiology) guidelines.

## 3 Results

The mean baseline LDL-C level was 117.1 ± 38.8 mg/dL. Follow-up LDL-C levels up to 4 years were between 73.7 and 77.9 mg/dL (Supplementary Table S1). The average LDL-C reduction rate for an initial 6 months was 34%. The patients were classified into four follow-up LDL-C strata: 20.2% (1,262/6,248) in LDL-C < 55 mg/dL, 26.5% (1,653/6,248) in LDL-C 55–69 mg/dL, 30.2% (1,890/6,248) in LDL-C 70–89 mg/dL, and 23.1% (1,443/6,248) in LDL-C ≥ 90 mg/dL. Baseline LDL-C levels and absolute LDL-C reduction according to these four strata are presented in Table 1.

**Table 1:** Baseline clinical characteristics and lipid lowering therapy according to absolute follow-up LDL-C strata.

Variable	Overall (*N* = 6,248)	LDL-C <55 (*N* = 1,262)	LDL-C 55–69 (*N* = 1,653)	LDL-C 70–89 (*N* = 1,890)	LDL-C ≥90 (*N* = 1,443)	*P* value*
Age, years	61.3 (12.1)	63.0 (11.6)	61.6 (12.0)	60.6 (11.9)	60.5 (12.6)	<0.001
Male sex	4,891 (78.3)	1,042 (82.6)	1,321 (79.9)	1,465 (77.5)	1,063 (73.7)	<0.001
ST-elevation myocardial infarction	3,273 (52.4)	678 (53.7)	884 (53.5)	1,004 (53.1)	707 (49.0)	0.033
Body mass index, kg/m^2^	24.3 (3.2)	24.0 (3.1)	24.2 (3.2)	24.4 (3.3)	24.5 (3.3)	<0.001
Hypertension	2,827 (45.2)	624 (49.4)	762 (46.1)	820 (43.4)	621 (43.0)	0.002
Diabetes mellitus	1,565 (25.0)	437 (34.6)	383 (23.2)	416 (22.0)	329 (22.8)	<0.001
Previous myocardial infarction	315 (5.0)	51 (4.0)	71 (4.3)	88 (4.7)	105 (7.3)	<0.001
Previous revascularization	471 (7.5)	85 (6.7)	104 (6.3)	133 (7.0)	149 (10.3)	<0.001
Previous cerebrovascular accident	280 (4.5)	66 (5.2)	67 (4.1)	85 (4.5)	62 (4.3)	0.480
Current smoker	2,842 (45.5)	548 (43.4)	734 (44.4)	859 (45.4)	701 (48.6)	0.015
Family history of premature coronary artery disease	53 (0.8)	8 (0.6)	15 (0.9)	14 (0.7)	16 (1.1)	0.535
Statin treatment						<0.001
No/low/moderate	4,194 (67.1)	782 (62.0)	1,038 (62.8)	1,316 (69.6)	1,058 (73.3)	
High	2,054 (32.9)	480 (38.0)	615 (37.2)	574 (30.4)	385 (26.7)	
Other lipid-lowering therapy						
Ezetimibe	321 (5.1)	58 (4.6)	71 (4.3)	120 (6.4)	72 (5.0)	0.030
Fibrate	35 (0.6)	7 (0.6)	7 (0.4)	11 (0.6)	10 (0.7)	0.794
LDL-C profiles						
Baseline LDL-C levels, mg/dL	117.1 (38.8)	99.8 (33.1)	112.0 (34.1)	122.3 (37.4)	131.3 (43.4)	<0.001
Absolute LDL-C reduction, mg/dL	42.0 (39.6)	54.4 (32.5)	49.8 (33.9)	43.5 (37.4)	20.5 (45.4)	<0.001

Values are presented as mean (SD) or number (%).

LDL-C, low-density lipoprotein cholesterol.

* *P*-values are derived from the chi-square test or Fisher's exact test for categorical variables, when appropriate, and from one-way analysis of variance test for continuous variables.

### 3.1 Baseline clinical characteristics by absolute follow-up LDL-C strata

Compared with patients with LDL-C ≥90 mg/dL, those with LDL-C <55 mg/dL were older, were more often male, were more likely to be ST-elevation myocardial infarction, and were more likely to have a low body mass index (Table 1). They were more likely to have a history of hypertension or diabetes mellitus but were less likely to have a history of myocardial infarction or revascularization and were less often current smokers. They were more likely to be treated with high-intensity statins. Other lipid-lowering therapies included ezetimibe in around 5% of patients and fibrate in less than 1%.

### 3.2 Clinical outcomes according to absolute follow-up LDL-C strata

**Figure 2:**
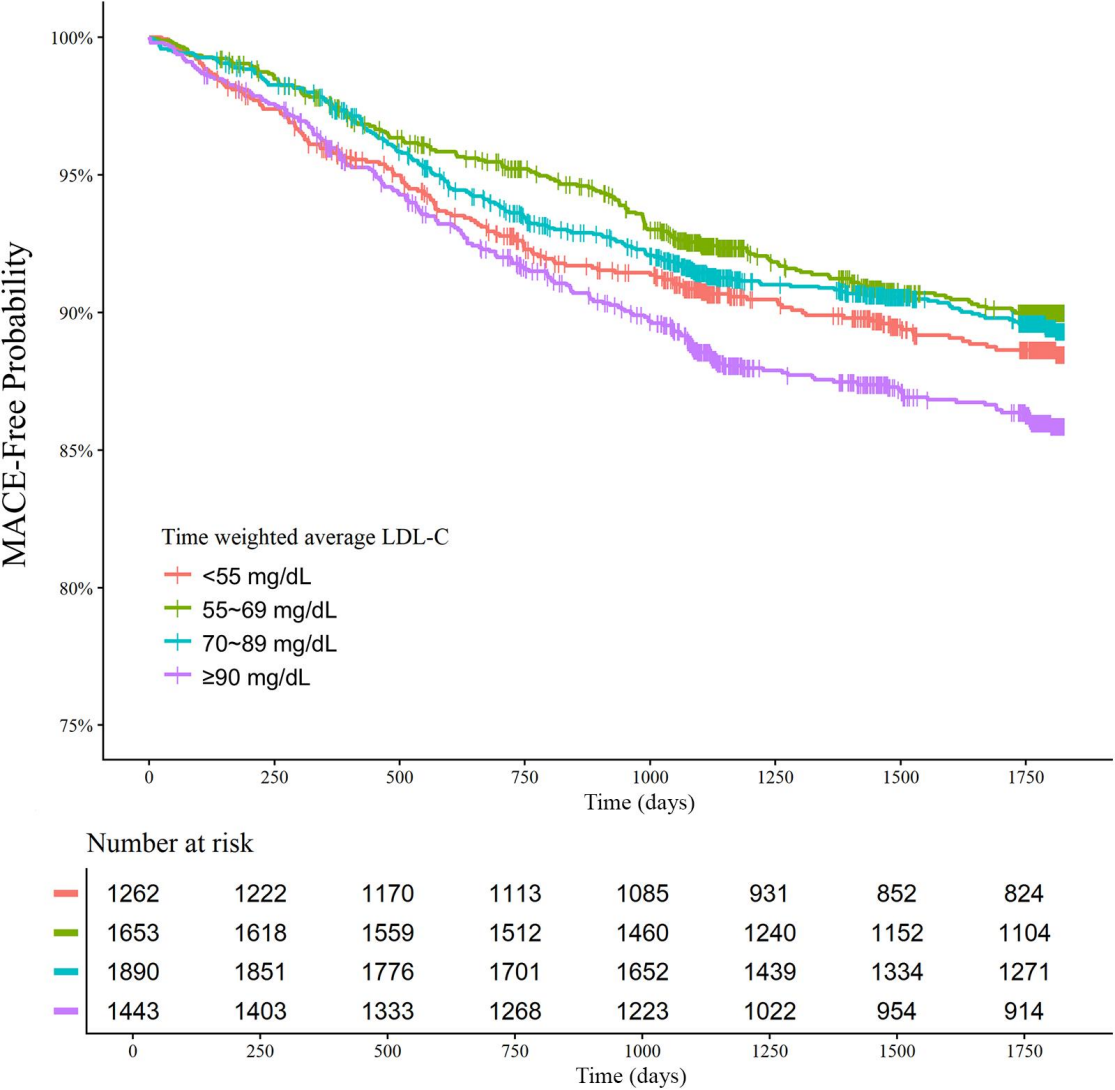
Absolute follow-up low-density lipoprotein cholesterol (LDL-C) strata and major adverse cardiovascular events (MACE) at 5 years. Kaplan–Meier curves for MACE over 5 years according to the follow-up LDL-C strata are shown. The MACE outcome was a composite of nonfatal stroke, nonfatal myocardial infarction, repeat revascularization, and all-cause death.

**Figure 3:**
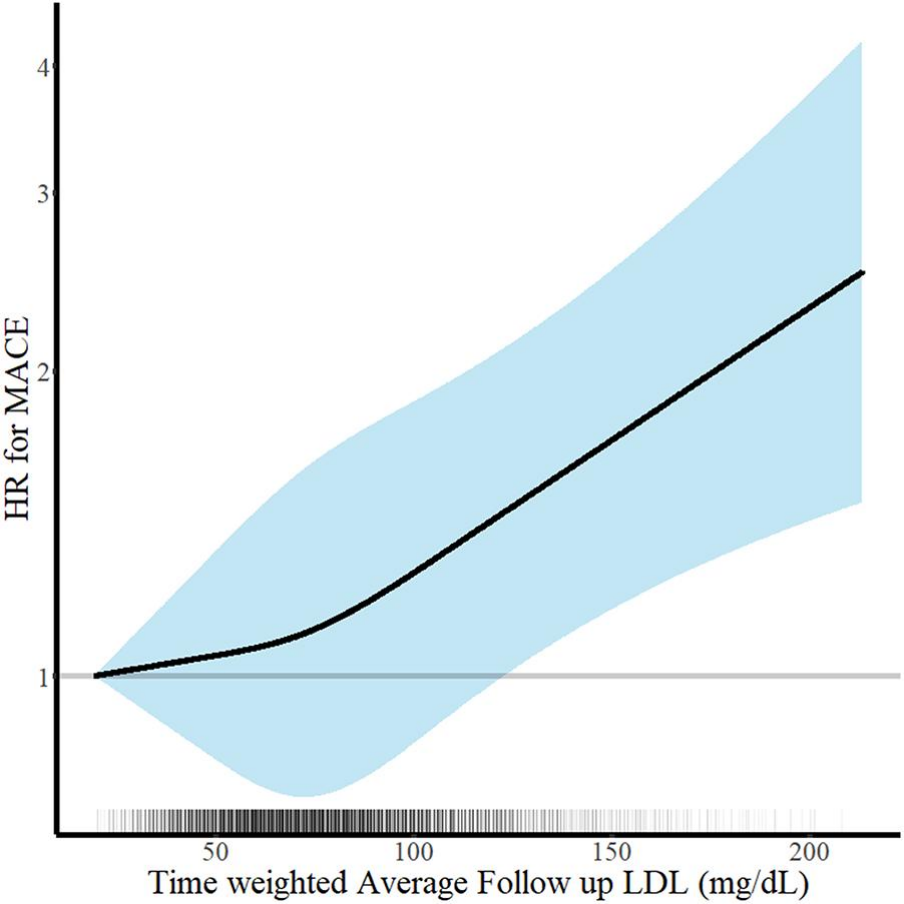
Continuous absolute follow-up low-density lipoprotein cholesterol (LDL-C) levels and risk of major adverse cardiovascular events (MACE) at 5 years. Hazard ratios for MACE at 5 years according to the continuous follow-up time-weighted average LDL-C levels are shown. Adjustments were performed for age, sex, body mass index, hypertension, diabetes mellitus, current smoker status, family history of premature coronary artery disease, previous myocardial infarction, previous myocardial revascularization, ST-elevation myocardial infarction diagnosis, and statins medication. Solid lines and shaded areas indicate hazard ratios (HRs) and 95% confidence intervals (CIs), respectively.

**Figure 4:**
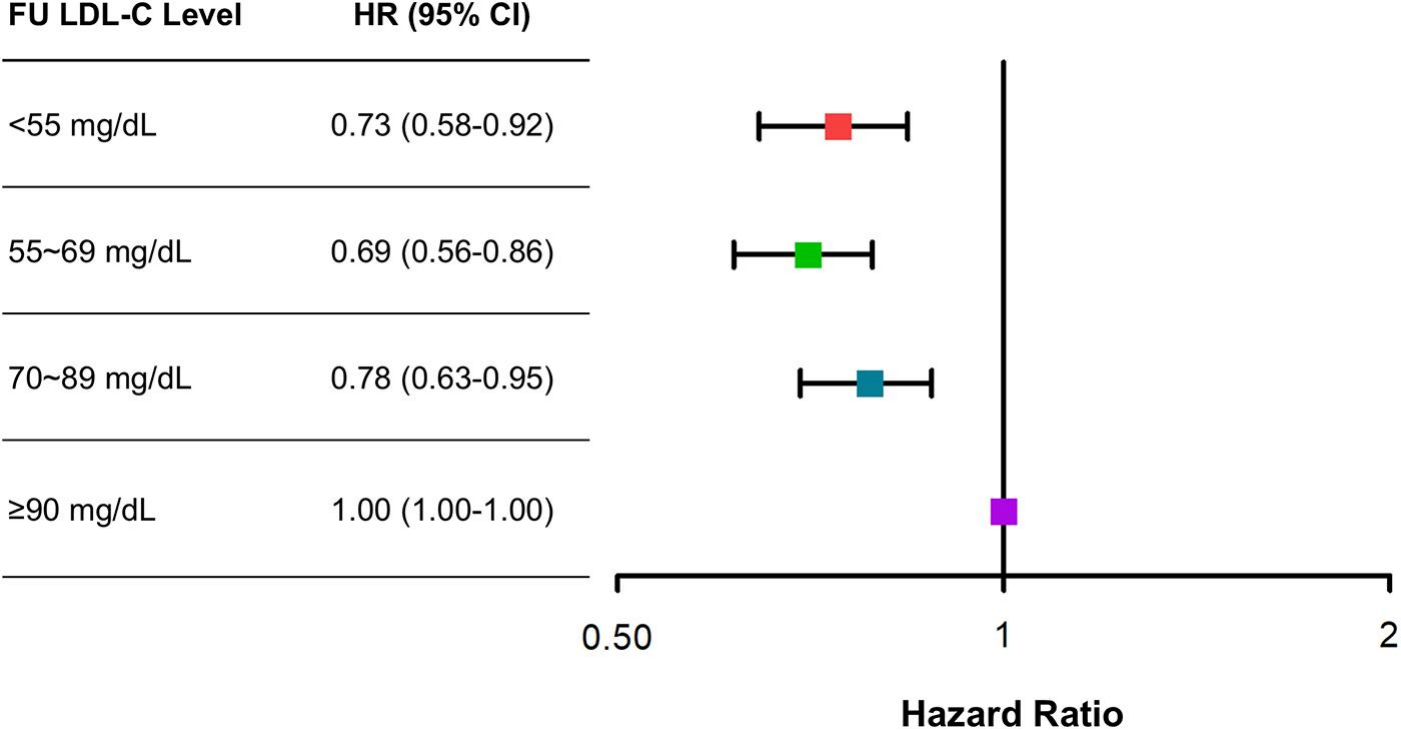
Adjusted risk of categorical absolute follow-up low-density lipoprotein cholesterol (LDL-C) strata for major adverse cardiovascular events (MACE) at 5 years. Adjusted hazard ratios (HRs) for MACE at 5 years with LDL-C ≥90 mg/dL as the reference. Cox regression analysis using the enter method was conducted.

The clinical outcomes were evaluated for up to 5 years (median 1,777 days; interquartile range 1,090–1,826 days). Among 6,248 patients with AMI, there were 312 all-cause deaths and 670 MACE at 5 years. There was a U-shaped trend of MACE incidence among the absolute follow-up LDL-C strata (136/1,262 [10.8%] in LDL-C <55 mg/dL vs. 154/1,653 [9.3%] in LDL-C 55–69 mg/dL vs. 189/1,890 [10.0%] in LDL-C 70–89 mg/dL vs. 191/1,443 [13.2%] in LDL-C ≥90 mg/dL for MACE, *P* = 0.003; and 82/1,262 [6.5%] vs. 70/1,653 [4.2%] vs. 73/1,890 [3.9%] vs. 87/1,443 [6.0%] for all-cause death, *P* < 0.001) (Table 2). Kaplan–Meier curves for MACE by the absolute follow-up LDL-C strata are shown in Figure 2. In a restricted cubic spline model, a J-shaped association was observed between absolute follow-up LDL-C levels and the risk of MACE (Figure 3). In a multivariable Cox time-to-event analysis with LDL-C level ≥90 mg/dL as the reference, the adjusted hazard ratios for MACE were 0.73 [95% confidence interval (CI), 0.58–0.92] in LDL-C < 55 mg/dL, 0.69 (95% CI, 0.56–0.86) in LDL-C 55–69 mg/dL, and 0.78 (95% CI, 0.63–0.95) in LDL-C 70–89 mg/dL (Figure 4). The results of univariable Cox time-to-event analysis are presented in Supplementary Table S2. There was a U-shaped association between absolute follow-up LDL-C levels and the risk of all-cause mortality in a restricted cubic spline model, with the lowest risk at an LDL-C level of 74.4 mg/dL (Supplementary Figure S1A). Kaplan–Meier curves for all-cause death by the absolute follow-up LDL-C strata are shown in Supplementary Figure S1B. The adjusted hazard ratios for all-cause death were 0.87 (95% CI, 0.63–1.18) in LDL-C <55 mg/dL, 0.67 (95% CI, 0.49–0.92) in LDL-C 55–69 mg/dL, and 0.67 (95% CI, 0.49–0.91) in LDL-C 70–89 mg/dL.

**Table 2:** Clinical outcomes according to absolute follow-up LDL-C strata.

Outcome	Overall (*N* = 6,248)	LDL-C <55 (*N* = 1,262)	LDL-C 55–69 (*N* = 1,653)	LDL-C 70–89 (*N* = 1,890)	LDL-C ≥90 (*N* = 1,443)	*P* value*
MACE	670 (10.7)	136 (10.8)	154 (9.3)	189 (10.0)	191 (13.2)	0.003
MACE individual endpoints						
Cardiac death	130 (19.4)	31 (22.8)	32 (20.8)	30 (15.9)	37 (19.4)	
Non-cardiac death	120 (17.9)	29 (21.3)	29 (18.8)	31 (16.4)	31 (16.2)	
Myocardial infarction	255 (38.1)	49 (36.0)	53 (34.4)	76 (40.2)	77 (40.3)	
Cerebrovascular event	142 (21.2)	24 (17.6)	36 (23.4)	44 (23.3)	38 (19.9)	
Coronary artery bypass grafting	23 (3.4)	3 (2.2)	4 (2.6)	8 (4.2)	8 (4.2)	
Death	312 (5.0)	82 (6.5)	70 (4.2)	73 (3.9)	87 (6.0)	<0.001
Death individual endpoints						
Cardiac death	162 (51.9)	42 (51.2)	37 (52.9)	37 (50.7)	46 (52.9)	
Non-cardiac death	150 (48.1)	40 (48.8)	33 (47.1)	36 (49.3)	41 (47.1)	

Values are presented as number (%).

LDL-C, low-density lipoprotein cholesterol; MACE, major adverse cardiac events.

* *P*-values are derived from the chi-square test or Fisher's exact test for categorical variables, when appropriate, and from one-way analysis of variance test for continuous variables.

### 3.3 Baseline clinical characteristics by percentage LDL-C reductions from baseline

Compared with patients with LDL-C reduction <0%, those with LDL-C reduction ≥50% were younger, were more often male, were more likely to have ST-elevation myocardial infarction, and were more likely to have a high body mass index (Table 3). They were less likely to have a history of hypertension, diabetes mellitus, previous myocardial infarction, previous revascularization, or previous cerebrovascular accident, but were more often current smokers. They were more likely to be treated with high-intensity statins.

**Table 3:** Baseline clinical characteristics and lipid lowering therapy according to percentage LDL-C reduction strata.

Variable	Overall (*N* = 6,248)	LDL-C reduction <0% (*N* = 876)	LDL-C reduction 0%–49% (*N* = 2,655)	LDL-C reduction ≥50% (*N* = 2,707)	*P* value*
Age, years	61.3 (12.1)	64.1 (12.2)	62.1 (11.9)	59.7 (11.9)	<0.001
Male sex	4,891 (78.3)	631 (72.0)	2,101 (78.8)	2,159 (79.8)	<0.001
ST-elevation myocardial infarction	3,273 (52.4)	374 (42.7)	1,420 (53.3)	1,479 (54.6)	<0.001
Body mass index, kg/m^2^	24.3 (3.2)	24.0 (3.3)	24.1 (3.3)	24.5 (3.1)	<0.001
Hypertension	2,827 (45.2)	478 (54.6)	1,286 (48.3)	1,063 (39.3)	<0.001
Diabetes mellitus	1,565 (25.0)	341 (38.9)	689 (25.9)	535 (19.8)	<0.001
Previous myocardial infarction	315 (5.0)	122 (13.9)	142 (5.3)	51 (1.9)	<0.001
Previous revascularization	471 (7.5)	199 (22.7)	200 (7.5)	72 (2.7)	<0.001
Previous cerebrovascular accident	280 (4.5)	76 (8.7)	125 (4.7)	79 (2.9)	<0.001
Current smoker	2,842 (45.5)	323 (36.9)	1,185 (44.5)	1,334 (49.3)	<0.001
Family history of premature coronary artery disease	53 (0.8)	6 (0.7)	26 (1.0)	21 (0.8)	0.619
Statin treatment					<0.001
No/low/moderate	4,194 (67.1)	736 (84.0)	1,960 (73.5)	1,498 (55.3)	
High	2,054 (32.9)	140 (16.0)	705 (26.5)	1,209 (44.7)	
Other lipid-lowering therapy					
Ezetimibe	321 (5.1)	46 (5.3)	131 (4.9)	144 (5.3)	0.792
Fibrate	35 (0.6)	11 (1.3)	13 (0.5)	11 (0.4)	0.022
LDL-C profiles					
Baseline LDL-C levels, mg/dL	117.1 (38.8)	75.1 (27.8)	102.1 (25.5)	145.5 (31.4)	<0.001
Absolute LDL-C reduction, mg/dL	75.0 (25.5)	96.8 (32.1)	74.7 (23.9)	68.3 (20.4)	<0.001

Values are presented as mean (SD) or number (%).

LDL-C, low-density lipoprotein cholesterol.

* *P*-values are derived from the chi-square test or Fisher's exact test for categorical variables, when appropriate, and from one-way analysis of variance test for continuous variables.

### 3.4 Clinical outcomes according to percentage LDL-C reductions from baseline

**Figure 5:**
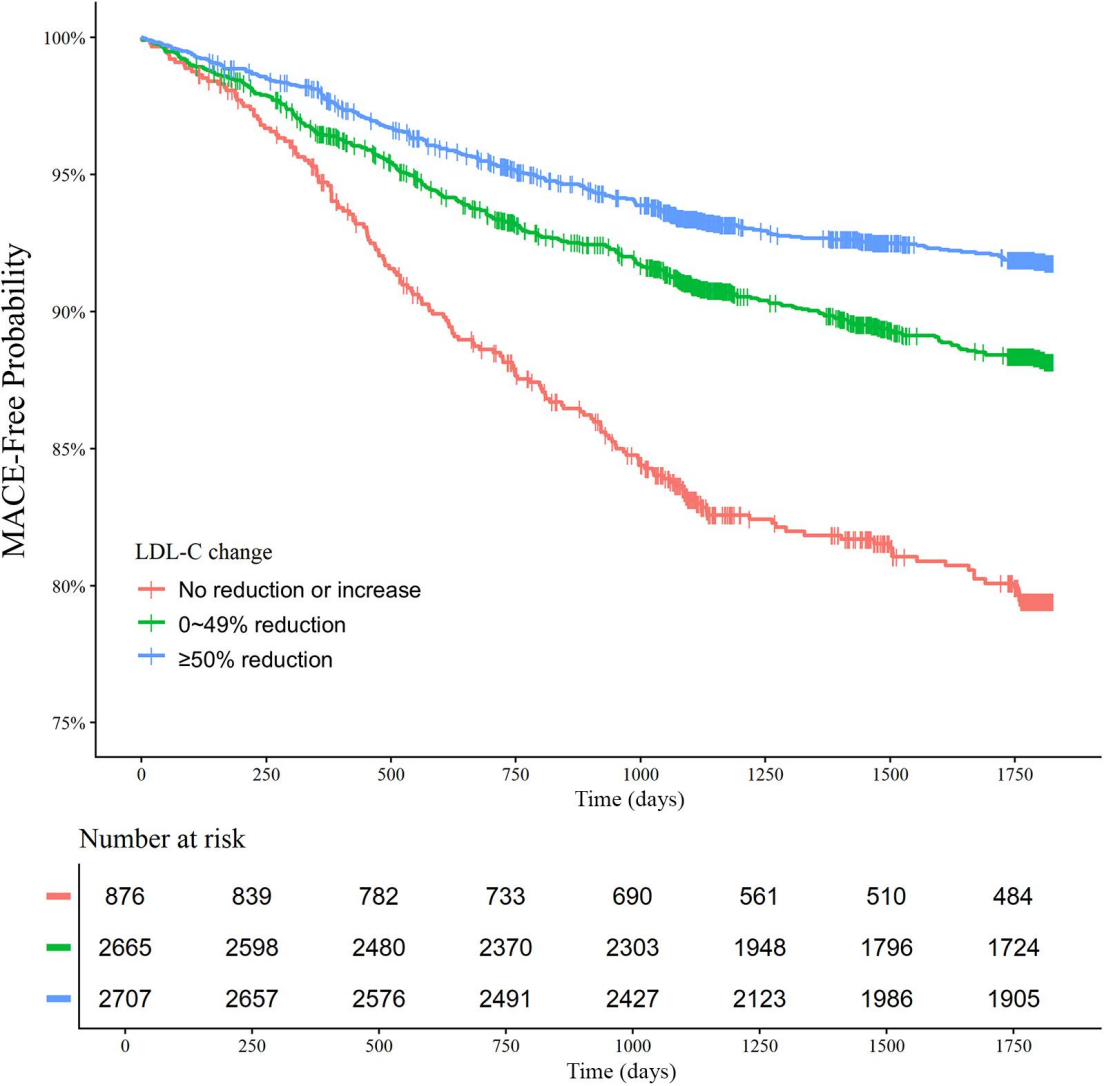
Low-density lipoprotein cholesterol (LDL-C) reduction strata and major adverse cardiovascular events (MACE) at 5 years. Kaplan–Meier curves for MACE over 5 years according to the LDL-C reduction strata are shown. The MACE outcome was a composite of nonfatal stroke, nonfatal myocardial infarction, repeat revascularization, and all-cause death.

**Figure 6:**
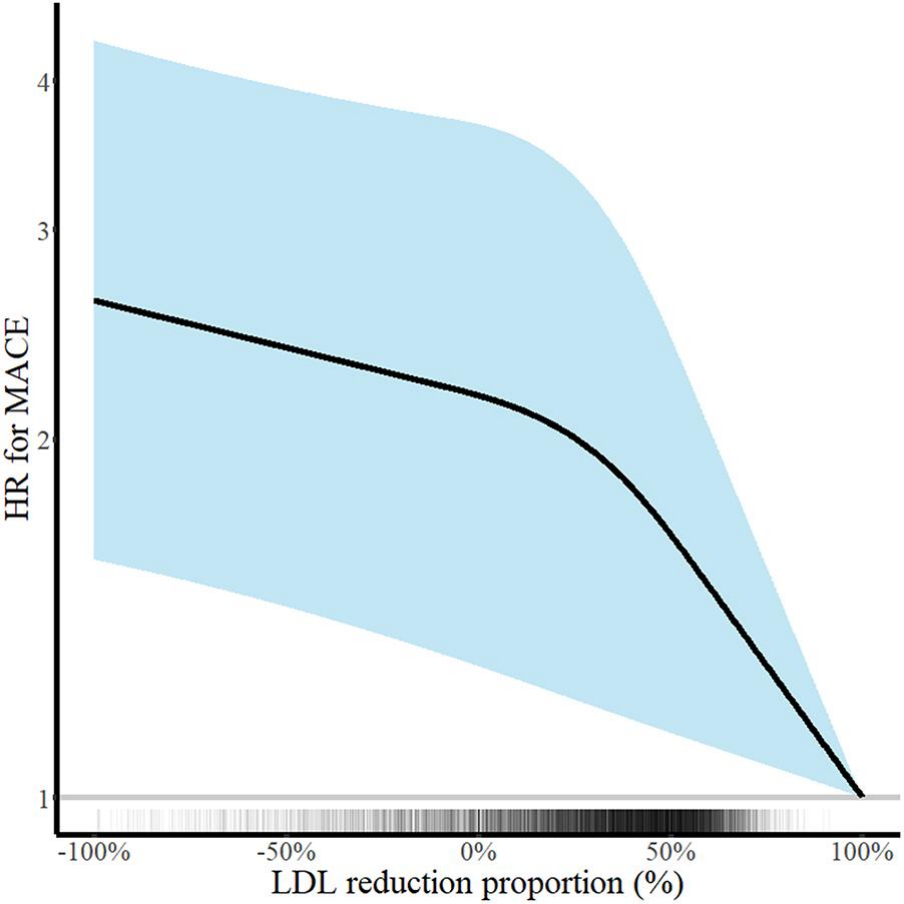
Continuous low-density lipoprotein cholesterol (LDL-C) reduction and risk of major adverse cardiovascular events (MACE) at 5 years. Hazard ratios for MACE at 5 years according to the percentage LDL-C reduction rate are shown. Adjustments were performed for age, sex, body mass index, hypertension, diabetes mellitus, current smoker status, family history of premature coronary artery disease, previous myocardial infarction, previous myocardial revascularization, ST-elevation myocardial infarction diagnosis, and statins medication. Solid lines and shaded areas indicate hazard ratios (HRs) and 95% confidence intervals (CIs), respectively.

**Figure 7:**
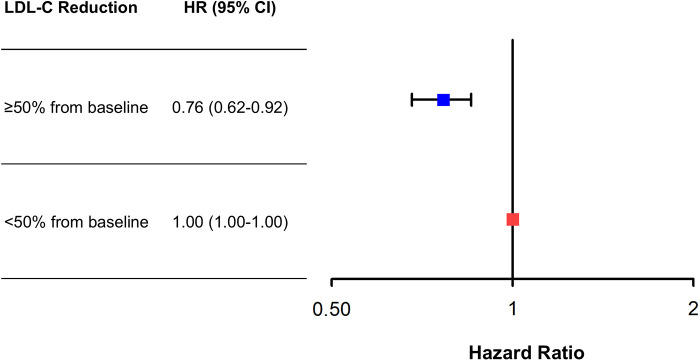
Adjusted risk of ≥50% low-density lipoprotein cholesterol (LDL-C) reduction from baseline vs. <50% reduction for major adverse cardiovascular events (MACE) at 5 years. Adjusted hazard ratios (HRs) of ≥50% LDL-C reduction from baseline for MACE at 5 years with <50% LDL-C reduction from baseline as the reference. Cox regression analysis using the enter method was conducted.

There was a decreasing trend of MACE incidence among the percentage LDL-C reductions from baseline strata (167/876 [19.1%] in LDL-C reduction <0% vs. 292/2,655 [11.0%] in LDL-C reduction 0%–49% vs. 211/2,707 [7.8%] in LDL-C reduction ≥50% for MACE, *P* < 0.001; and 82/876 [9.4%] vs. 152/2,655 [5.7%] vs. 78/2,707 [2.9%] for all-cause death, *P* < 0.001) (Table 4). Kaplan–Meier curves for MACE by the categorical percentage LDL-C reduction strata over 5 years demonstrated that patients with higher LDL-C reduction from baseline had a lower risk of MACE (Figure 5). For analysis of the LDL-C reduction rate from baseline in a restricted cubic spline model, the greater the reduction, the lower the risk of MACE (Figure 6). In a multivariable Cox time-to-event analysis with LDL-C < 50% reduction from baseline as the reference, ≥50% LDL-C reduction from baseline was independently associated with a lower incidence of MACE (adjusted hazard ratio 0.76; 95% CI, 0.62–0.92) (Figure 7). The results of univariable Cox time-to-event analysis are presented in Supplementary Table S3. A restricted cubic spline model and Kaplan–Meier curves for the association between percentage LDL-C reduction and all-cause death are shown in Supplementary Figure S2. In a multivariable Cox time-to-event analysis with LDL-C < 50% reduction from baseline as the reference, ≥50% LDL-C reduction from baseline was independently associated with a lower incidence of deaths (adjusted hazard ratio 0.67; 95% CI, 0.49–0.91).

**Table 4:** Clinical outcomes according to percentage LDL-C reduction strata.

Outcome	Overall (*N* = 6,248)	LDL-C reduction <0% (*N* = 876)	LDL-C reduction 0%–49% (*N* = 2,655)	LDL-C reduction ≥50% (*N* = 2,707)	*P* value*
MACE	670 (10.7)	167 (19.1)	292 (11.0)	211 (7.8)	<0.001
MACE individual endpoints					
Cardiac death	130 (19.4)	39 (23.4)	61 (20.9)	30 (14.2)	
Non-cardiac death	120 (17.9)	28 (16.8)	63 (21.6)	29 (13.7)	
Myocardial infarction	255 (38.1)	57 (34.1)	104 (35.6)	94 (44.5)	
Cerebrovascular event	142 (21.2)	39 (23.4)	52 (17.8)	51 (24.2)	
Coronary artery bypass grafting	23 (3.4)	4 (2.4)	12 (4.1)	7 (3.3)	
Death	312 (5.0)	82 (9.4)	152 (5.7)	78 (2.9)	<0.001
Death individual endpoints					
Cardiac death	162 (51.9)	47 (57.3)	75 (49.3)	40 (51.3)	
Non-cardiac death	150 (48.1)	35 (42.7)	77 (50.7)	38 (48.7)	

Values are presented as number (%).

LDL-C, low-density lipoprotein cholesterol; MACE, major adverse cardiac events.

* *P*-values are derived from the chi-square test or Fisher's exact test for categorical variables, when appropriate, and from one-way analysis of variance test for continuous variables.

### 3.5 Subgroup analysis

When stratified by sex, the associations between clinical outcomes and absolute follow-up LDL-C levels vs. percentage LDL-C reduction had somewhat differing slopes in some parts but generally similar trends (Supplementary Figures S3 and S4).

## 4 Discussion

In a nationwide Korean cohort study with 5 years of clinical follow-up, we investigated the association between absolute value vs. percentage reduction of follow-up LDL-C levels and clinical outcomes at 5 years. For the analysis of absolute categorical follow-up LDL-C strata, there was a U-shaped trend of MACE incidence. In the analysis of continuous follow-up LDL-C levels using an adjusted cubic spline model, there was a J-shaped association between the LDL-C levels and MACE. For the analysis of the percentage LDL-C reduction from baseline, the greater the reduction rate, the lower the risk of MACE. In a multivariable Cox time-to-event analysis, a ≥50% LDL-C reduction from baseline was independently associated with a decreased incidence of MACE.

Recent American and European guidelines on dyslipidemia emphasize lowering LDL-C levels for secondary prevention in patients at very high risk of atherosclerotic cardiovascular disease, including coronary artery disease (5, 6). However, global studies on LDL-C target achievement in real-world practice have used absolute follow-up LDL-C levels such as <70 mg/dL, which have revealed poor LDL-C target achievement rates (7–12). Barriers to achieving guideline-recommended LDL-C targets can exist at the patient, physician, and healthcare system levels (5). Recently, a survey in Korea on the optimal LDL-C targets for AMI patients demonstrated that a considerable proportion of physicians prefer to use only absolute follow-up LDL-C targets of “<70 mg/dL or <55 mg/dL” without considering percentage LDL-C reduction from baseline (13). In a real-world setting, using a percent LDL-C reduction as a target can be time-consuming because baseline LDL-C levels must be determined. Guidelines state that the term “baseline” refers to the LDL-C level when not taking any LDL-C-lowering drugs. When taking LDL-C-lowering drugs, the baseline LDL-C levels should be estimated based on the average LDL-C-lowering efficacy of the given medication (6).

There has been a bunch of evidence that intensive lipid-lowering therapy either slows disease progression or promotes plaque regression by modifying both the quantity and the composition of coronary atherosclerotic plaques (16). However, the degree of atheroma regression shown in concomitant imaging trials appeared more modest as compared to the magnitude of clinical benefit accrued from high-intensity statin therapy. Two landmark randomized trials of the proprotein convertase subtilisin/kexin 9 inhibitors, the HUYGENS and the PACKMAN-AMI, have demonstrated the additional effects of achieving very low levels of LDL-C on high-risk plaque features, including fibrous cap thickness and large lipid accumulation, beyond its size (17, 18). Recent meta-analysis assessing the effect of lipid-lowering therapy on coronary artery plaque in East Asia population revealed that the lowest levels of LDL-C (≤55 mg/dL) were associated with the greatest decrease (–1.56%, 95% CI: −2.20% to – 0.92%; *I*^2^ = 0%) of percent atheroma volume when compared with levels in the range of 55–70, 70–100, and 100–130 mg/dL (19). This finding suggests that intensive lipid-lowering therapy with a lower target LDL-C, especially to <55 mg/dL, would be beneficial for atherosclerosis treatment. However, most randomized clinical trials of intracoronary imaging included patients with LDL-C levels of at least 70 or 100 mg/dL and were inherently different from a real-world setting in terms of the patient population.

The present study demonstrated a U-shaped relationship between absolute follow-up LDL-C levels and the risk of MACE. This might be partly explained by the cholesterol paradox, which has been demonstrated in observational cohort studies. Patients with low follow-up LDL-C levels were more likely to have low baseline LDL-C levels. Several previous studies have reported that very low baseline LDL-C levels are associated with poor survival in patients with or without cardiovascular disease (20–23). It has been suggested that low cholesterol levels are associated with older age, inadequate nutritional status, or frailty (20, 22–24). Iribarren et al. analyzed total cholesterol changes in 5,941 middle-aged Japanese-Americans over a 6-year period and related these data to subsequent 16-year mortality data and suggested that falling total cholesterol levels occur before the development of certain cancers or non-cardiovascular diseases (particularly liver disease) (21). A prespecified analysis of The Improved Reduction of Outcomes: Vytorin Efficacy International Trial (IMPROVE-IT) demonstrated that patients with very low LDL-C levels of <30 mg/dL at 1 month after enrollment had a higher incidence of malignancy during the 5 years follow-up compared with those with LDL-C levels of >70 mg/dL (25). A recent multicenter Korean AMI registry involving 5,532 patients with 5 years of follow-up demonstrated that a baseline LDL-C level <70 mg/dL was independently associated with an increased incidence of cardiovascular death and MACE after discharge (26). Furthermore, a meta-analysis involving ≥130,000 patients revealed that more intensive LDL-C reduction compared with less intensive reduction was associated with a greater reduction in total and cardiovascular mortality risk in patients with higher baseline LDL-C levels, but not in those with baseline LDL-C levels of <100 mg/dL (27). These findings suggest that we use an integrated model to predict the net clinical benefit from lipid-lowering therapy, considering each patient's baseline LDL-C levels and risk profile.

In the present study, the greater the LDL-C reduction from baseline, the lower the MACE incidence. Moreover, a ≥50% LDL-C reduction from baseline was independently associated with a decreased incidence of MACE after multivariable adjustments. The benefit of lipid-lowering therapy on clinical outcomes is proportionate to the magnitude of LDL-C lowering in patients with atherosclerotic cardiovascular disease (28). Recent dyslipidemia guidelines recommend achieving ≥50% LDL-C reduction from baseline as the first target when implementing lipid-lowering therapy in patients with atherosclerotic cardiovascular disease (5, 6). In a nationwide prospective Korean cohort study involving 1,305 patients with AMI with a 2-year clinical follow-up, compared with <50% LDL-C reduction from baseline at 1 year, patients with ≥50% LDL-C reduction had a 47% risk reduction in MACE. However, compared with LDL-C levels ≥70 mg/dL at 1 year, patients with LDL-C levels <70 mg/dL had a similar risk of MACE (29). Schubert et al. analyzed 40,607 patients with AMI with a median 3.78 years follow-up in a real-world cohort and demonstrated that larger early LDL-C reductions after AMI events were associated with better cardiovascular outcomes and all-cause mortality (30).

To date, no confirmatory clinical trial has been conducted to compare the efficacy and safety of two targets: an absolute follow-up LDL-C level and a percentage LDL-C reduction. Although a meta-analysis indicated that LDL-C lowering may not be beneficial for all-cause and cardiovascular mortality end points in trials with more than 50% LDL-C reduction and in trials with low baseline LDL-C levels (31), substantial evidence supports the notion that greater LDL-C reductions lead to better clinical outcomes in patients with acute coronary syndrome (2). Furthermore, the Further Cardiovascular Outcomes Research With Proprotein Convertase Subtilisin/Kexin Type 9 Inhibition in Subjects With Elevated Risk-Open Label Extension study recently demonstrated that achieving lower LDL-C levels long-term, down to <20 mg/dL (<0.5 mmol/L), was associated with the best cardiovascular outcomes with no significant safety concerns in patients with atherosclerotic cardiovascular disease (32). Taken together, the main finding of the present study and the evidence from previous literature suggests that a percentage reduction in LDL-C would be a better marker after LDL-C-lowering therapy than an absolute follow-up LDL-C target, such as 70 or 55 mg/dL, in a real-world setting. For example, if one patient diagnosed with AMI has LDL-C levels of 70 mg/dL at baseline, just having LDL-C levels of 50 mg/dL may not mean that a patient would have a chance to show the best clinical outcomes. We should consider achieving LDL-C levels of less than 35 mg/dL in this patient, which is at least a 50% reduction from baseline levels.

The findings of the present study should be considered with the following limitations. First, the KAMIR-NIH-LIPID study has a retrospective nature, where additional clinical data regarding lipid management and clinical outcomes between the 3- and 5-year follow-up periods were collected retrospectively based on the electronic medical records between May 2022 and August 2023. Therefore, the possibility of missing data, including clinical outcomes, exists. Second, due to the nature of the present study, which compared the percentage LDL-C reductions from baseline and absolute follow-up levels after AMI, there are inherent issues about selection and immortal time biases. centers from KAMIR-NIH tended to be larger-volume teaching centers. Thus, practice patterns and clinical outcomes may not be generalized to the average hospital. Third, of the 13,188 patients with AMI, only 6,990 (53%) were included in the final analysis, resulting in attenuated internal validity. A comparison of baseline characteristics and clinical outcomes between analyzed and excluded patients is provided in Supplementary Table S4. Nonetheless, the patients analyzed in the study may present a similar population where physicians consider lipid-lowering strategy modifications using follow-up LDL-C levels in real-world practice. Finally, during the KAMIR-NIH period between 2011 and 2015, non-statin LDL-C-lowering drugs, including ezetimibe and proprotein convertase subtilisin/kexin type 9 inhibitors, generally were not considered in real-world practice in Korea.

In conclusion, this nationwide Korean registry involving 6,249 AMI patients with a 5-year clinical follow-up demonstrated that the greater the LDL-C reduction from baseline, the lower the incidence of MACE, while there was no clear decreasing trend in the risk of MACE when absolute follow-up LDL-C levels were lowered from around 70 mg/dL. Our study suggests that the percentage reduction in LDL-C from baseline may be a better marker than absolute follow-up LDL-C levels for LDL-C-lowering therapy in patients with AMI even if it's sometimes tricky to find out the baseline LDL-C levels of patients for lipid management after AMI in a real-world setting. Further studies are needed to clarify this issue.

## Data Availability

The anonymized data that support the results of this study can be made available upon reasonable request.
