# Supplementary material for: Long-term outcomes according to absolute value vs. percentage reduction in low-density lipoprotein cholesterol levels after acute myocardial infarction
Source: Front Cardiovasc Med. 2025 Dec 5;12:1653447. doi: 10.3389/fcvm.2025.1653447 (PMC12714945; doi:10.3389/fcvm.2025.1653447)
Supplement: Supplementary file 1 [file Datasheet1.pdf]

Supplementary Material

Supplement to: Cho KH, Yang JH, Lee SY, et al. **Long-Term Outcomes According to Absolute Value Versus Percentage Reduction in Low-Density Lipoprotein Cholesterol Levels After Acute Myocardial Infarction**

Table of Contents

Supplemental Methods

- KAMIR-NIH participating institutions and principal investigators 2
- KAMIR inclusion and exclusion criteria 3

Table S1. Mean LDL-C levels at baseline and over a 4-year follow-up period. 4

Table S2. Results of the Cox proportional hazard models applied to assess correlates of 5-year MACE including categorical follow-up low-density lipoprotein cholesterol strata. 5

Table S3. Results of the Cox proportional hazard models applied to assess correlates of 5-year MACE including ≥50% low-density lipoprotein cholesterol reduction from baseline. 6

Table S4. Comparison of baseline characteristics and clinical outcomes between analyzed and excluded patients. 7

Figure S1. Absolute follow-up low-density lipoprotein cholesterol (LDL-C) levels and all-cause death over 5 years. 8

Figure S2. Low-density lipoprotein cholesterol (LDL-C) reduction from baseline and all-cause death over 5 years. 9

Figure S3. Continuous follow-up low-density lipoprotein cholesterol (LDL-C) levels and cardiovascular (CV) outcomes stratified by sex. 10

Figure S4. Percentage low-density lipoprotein cholesterol (LDL-C) reduction rate and cardiovascular (CV) outcomes stratified by sex. 11

Supplemental methods

KAMIR-NIH participating institutions (Number of Patients) and investigators

1. Chonnam National University Hospital (3,182 patients), Gwangju, Korea; Myung Ho Jeong, principal investigator
2. Seoul National University Hospital (429 patients), Seoul, Korea; Hyo Soo Kim
3. Sungkyunkwan University, Seoul Samsung Medical Center (448 patients), Seoul, Korea; Joo-Young Hahn
4. The Catholic University of Korea, Seoul St. Mary's Hospital (450 patients), Seoul, Korea; Kiyuk Chang
5. Korea University Guro Hospital (454 patients), Seoul, Korea; Seung-Woon Rha
6. Kyungpook National University Hospital (1,250 patients), Daegu, Korea; Jang Hoon Lee
7. Kyung Hee University Hospital at Gangdong (219 patients), Seoul, Korea; Jin Man Cho
8. Pusan National University Hospital (518 patients), Busan, Korea; Kwang Soo Cha
9. Yeungnam University Hospital (792 patients), Daegu, Korea; Jong Seon Park
10. Wonju Severance Christian Hospital (235 patients), Wonju, Korea; Jang-Young Kim
11. Chonbuk National University Hospital (717 patients), Jeonju, Korea; Sang-Rok Lee
12. Jeju National University Hospital (130 patients), Jeju, Korea; Seung Jae Joo
13. Seoul National University Bundang Hospital (870 patients), Seongnam, Korea; Ju-Seung Kwun
14. Keimyung University Dongsan Medical Center (730 patients), Daegu, Korea; Seung Ho Hur
15. Chungnam National University Hospital (955 patients), Daejeon, Korea; Jin-Ok Jeong
16. Chungbuk National University Hospital (452 patients), Cheongju, Korea; Kyung Kook Hwang
17. Inje University Haeundae Paik Hospital (244 patients), Busan, Korea; Doo Il Kim
18. Wonkwang University Hospital (589 patients), Iksan, Korea; Seok Kyu Oh
19. Gachon University Gil Medical Center (392 patients), Incheon, Korea; Seung Hwan Han
20. Gyeongsang National University Hospital (606 patients), Jinju, Korea; Jin Yong Hwang

KAMIR-NIH inclusion and exclusion criteria

Patients diagnosed with acute myocardial infarction based on the universal definition were eligible for inclusion. We excluded (1) patients with in-hospital acute myocardial infarction related to other procedures or treatments (e.g., surgery) and (2) those who refused to provide informed consent.

Table S1. Mean LDL-C levels at baseline and over a 4-year follow-up period.

**Period**	**Baseline**	**6 months**	**12 months**	**18 months**	**24 months**	**30 months**	**36 months**	**42 months**	**48 months**
No. of patients	12569	3666	5958	2457	3909	2323	3974	2947	2767
Mean	111.5	73.7	75.6	77.1	77.7	76.6	77.9	76.6	75.4
SD	39.4	26.7	26.4	27.6	27.1	26.2	35.5	25.9	25.4

LDL-C = low-density lipoprotein cholesterol; SD = standard deviation.

Table S2. Results of the Cox proportional hazard models applied to assess correlates of 5-year MACE including categorical follow-up low-density lipoprotein cholesterol (LDL-C) strata.

	Odds ratio (95% confidence interval)			
	**No adjustment**	***P* value**	**Adjustment**	***P* value[a]**
LDL-C ≥90 mg/dL	–	–	–	–
LDL-C 70~89 mg/dL	0.74 (0.60-0.90)	0.003	0.78 (0.63-0.95)	0.014
LDL-C 55~69 mg/dL	0.68 (0.55-0.85)	<0.001	0.69 (0.56-0.86)	0.001
LDL-C <55 mg/dL	0.81 (0.65-1.01)	0.058	0.73 (0.58-0.92)	0.006
Age, years	1.05 (1.04-1.06)	<0.001	1.04 (1.03-1.05)	<0.001
Male sex	0.59 (0.50-0.70)	<0.001	0.97 (0.78-1.19)	0.744
Body mass index, kg/m^2	0.92 (0.90-0.95)	<0.001	0.96 (0.94-0.99)	0.003
Hypertension	1.74 (1.49-2.03)	<0.001	1.19 (1.01-1.40)	0.035
Diabetes mellitus	2.19 (1.87-2.55)	<0.001	1.77 (1.50-2.07)	<0.001
Current smoking	1.82 (1.53-2.16)	<0.001	0.99 (0.80-1.23)	0.918
Family history of premature coronary artery disease	1.44 (0.72-2.90)	0.303	1.97 (0.98-3.97)	0.058
Previous history of myocardial infarction	2.73 (2.16-3.45)	<0.001	1.14 (0.82-1.57)	0.438
Previous history of myocardial revascularization	3.15 (2.60-3.83)	<0.001	2.18 (1.66-2.86)	<0.001
STEMI diagnosis	0.71 (0.61-0.83)	<0.001	0.86 (0.74-1.01)	0.064
Statins medication	0.71 (0.59-0.84)	<0.001	0.86 (0.72-1.03)	0.094

STEMI, ST elevation myocardial infarction.

[a]Cox regression analysis using the enter method was performed.

Table S3. Results of the Cox proportional hazard models applied to assess correlates of 5-year MACE including ≥50% low-density lipoprotein cholesterol (LDL-C) reduction from baseline.

	Odds ratio (95% confidence interval)			
	**No adjustment**	***P* value**	**Adjustment**	***P* value**[a]
< 50% LDL-C reduction	-	-	-	-
≥ 50% LDL-C reduction	0.62 (0.51-0.75)	<0.001	0.76 (0.62-0.92)	0.006
Age, years	1.05 (1.04-1.06)	<0.001	1.04 (1.03-1.05)	<0.001
Male sex	0.59 (0.50-0.70)	<0.001	0.92 (0.75-1.14)	0.458
Body mass index, kg/m^2	0.92 (0.90-0.95)	<0.001	0.96 (0.94-0.99)	0.004
Hypertension	1.74 (1.49-2.03)	<0.001	1.18 (1.00-1.39)	0.049
Diabetes mellitus	2.19 (1.87-2.55)	<0.001	1.75 (1.49-2.05)	<0.001
Current smoking	1.82 (1.53-2.16)	<0.001	0.97 (0.78-1.21)	0.790
Family history of premature coronary artery disease	1.44 (0.72-2.90)	0.303	2.01 (1.00-4.06)	0.050
Previous history of myocardial infarction	2.73 (2.16-3.45)	<0.001	1.14 (0.83-1.58)	0.412
Previous history of myocardial revascularization	3.15 (2.60-3.83)	<0.001	2.14 (1.63-2.81)	<0.001
STEMI diagnosis	0.71 (0.61-0.83)	<0.001	0.86 (0.73-1.00)	0.051
Statins medication	0.71 (0.59-0.84)	<0.001	0.87 (0.73-1.04)	0.129

STEMI, ST elevation myocardial infarction.

[a]Cox regression analysis using the enter method was performed.

Table S4. Comparison of baseline characteristics and clinical outcomes between analyzed and excluded patients.

	Analyzed patients (N = 6248)	Non-analyzed patients (N = 7414)	P value*
Age, years	61.3 (12.1)	66.4 (12.8)	<0.001
Male sex	4891 (78.3)	5130 (69.2)	<0.001
ST-elevation myocardial infarction	3273 (52.4)	3093 (44.6)	<0.001
Body mass index, kg/m^2	24.3 (3.2)	23.7 (3.4)	<0.001
Hypertension	2827 (45.2)	4178 (56.4)	<0.001
Diabetes mellitus	1565 (25.0)	2362 (31.9)	<0.001
Previous myocardial infarction	315 (5.0)	787 (10.6)	<0.001
Previous revascularization	471 (7.5)	1149 (15.5)	<0.001
Current smoker	2217 (35.5)	3274 (46.4)	<0.001
Family history of premature coronary artery disease	53 (0.8)	51 (0.7)	0.500
Baseline LDL-C levels, mg/dL	117.1 (38.8)	106.0 (39.3)	<0.001
High-intensity statins	2054 (32.9)	1820 (24.6)	<0.001
Clinical outcomes			
MACE	670 (10.7)	1420 (19.2)	<0.001
All-cause death	312 (5.0)	1015 (13.7)	<0.001

Values are presented as mean (SD) or number (%). *P-values are derived from the chi-square test for categorical variables and independent t-test for continuous variables. LDL-C = low-density lipoprotein cholesterol; MACE = major adverse cardiovascular event

Figure S1. Absolute follow-up low-density lipoprotein cholesterol (LDL-C) levels and all-cause death over 5 years.

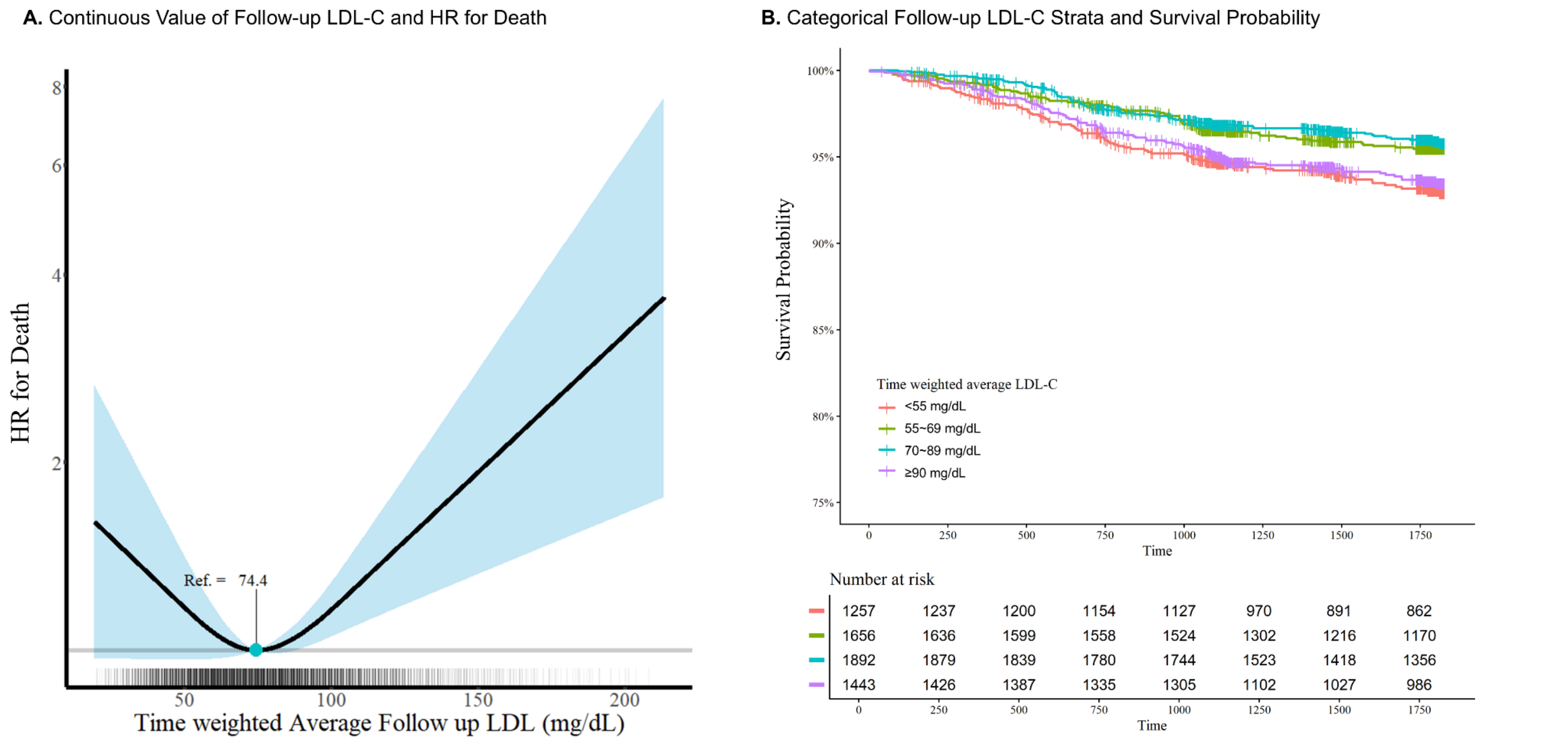

(A) Hazard ratios for all-cause death at 5 years according to the continuous follow-up time-weighted average LDL-C levels are shown. Adjustments were performed for age, sex, body mass index, hypertension, diabetes mellitus, current smoker status, family history of premature coronary artery disease, previous myocardial infarction, previous myocardial revascularization, ST-elevation myocardial infarction diagnosis, and statins medication. (B) Kaplan–Meier curves for all-cause death over 5 years according to the categorical follow-up LDL-C strata are shown.

Figure S2. Low-density lipoprotein cholesterol (LDL-C) reduction from baseline and all-cause death over 5 years.

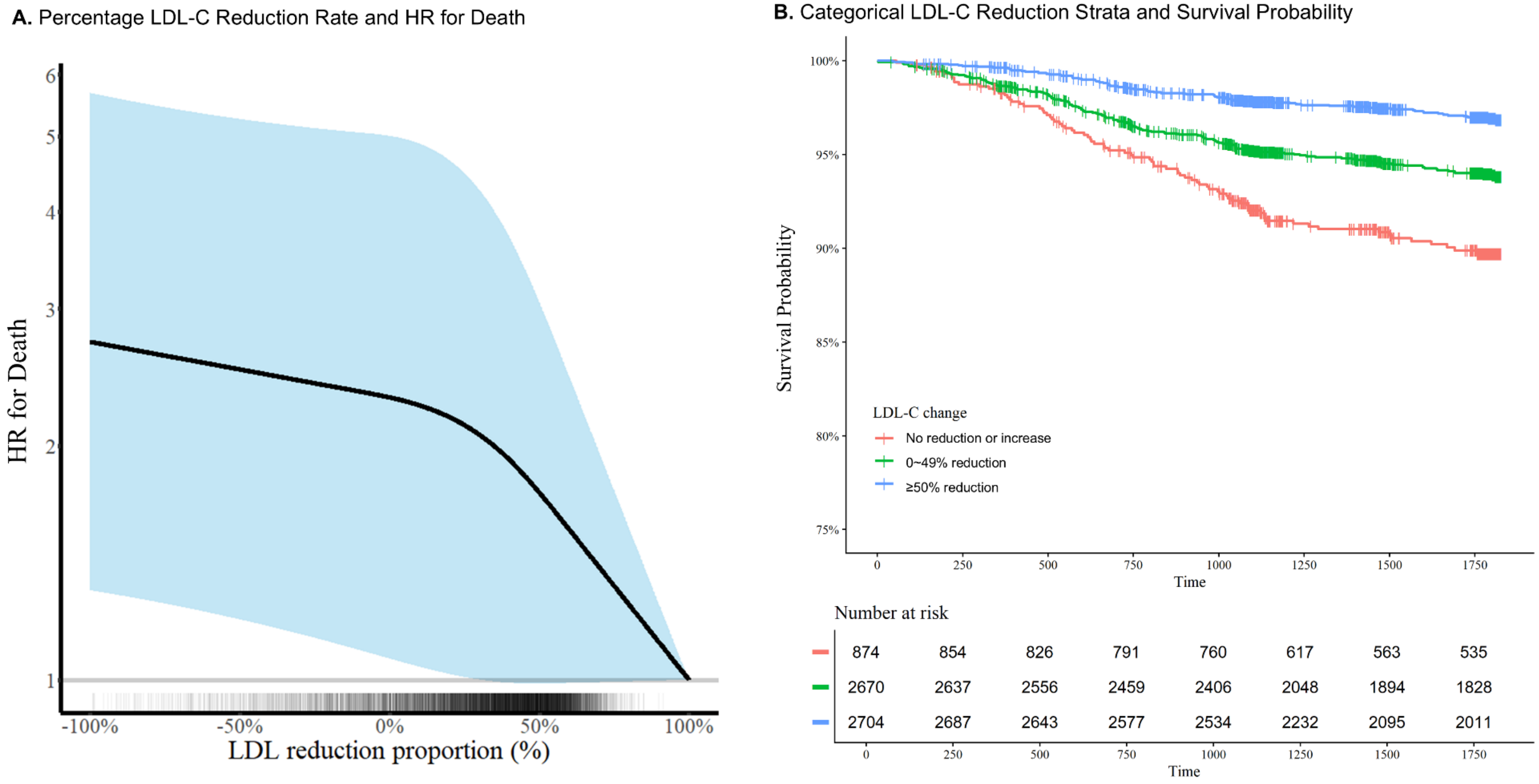

(A) Hazard ratios for all-cause death at 5 years according to the percentage LDL-C reduction rate are shown. Adjustments were performed for age, sex, body mass index, hypertension, diabetes mellitus, current smoker status, family history of premature coronary artery disease, previous myocardial infarction, previous myocardial revascularization, ST-elevation myocardial infarction diagnosis, and statins medication. (B) Kaplan–Meier curves for all-cause death over 5 years according to the percentage LDL-C reduction strata are shown.

Figure S3. Continuous absolute follow-up low-density lipoprotein cholesterol (LDL-C) levels and cardiovascular (CV) outcomes at 5 years stratified by sex. (Men, n = 4891; Women, n = 1357)

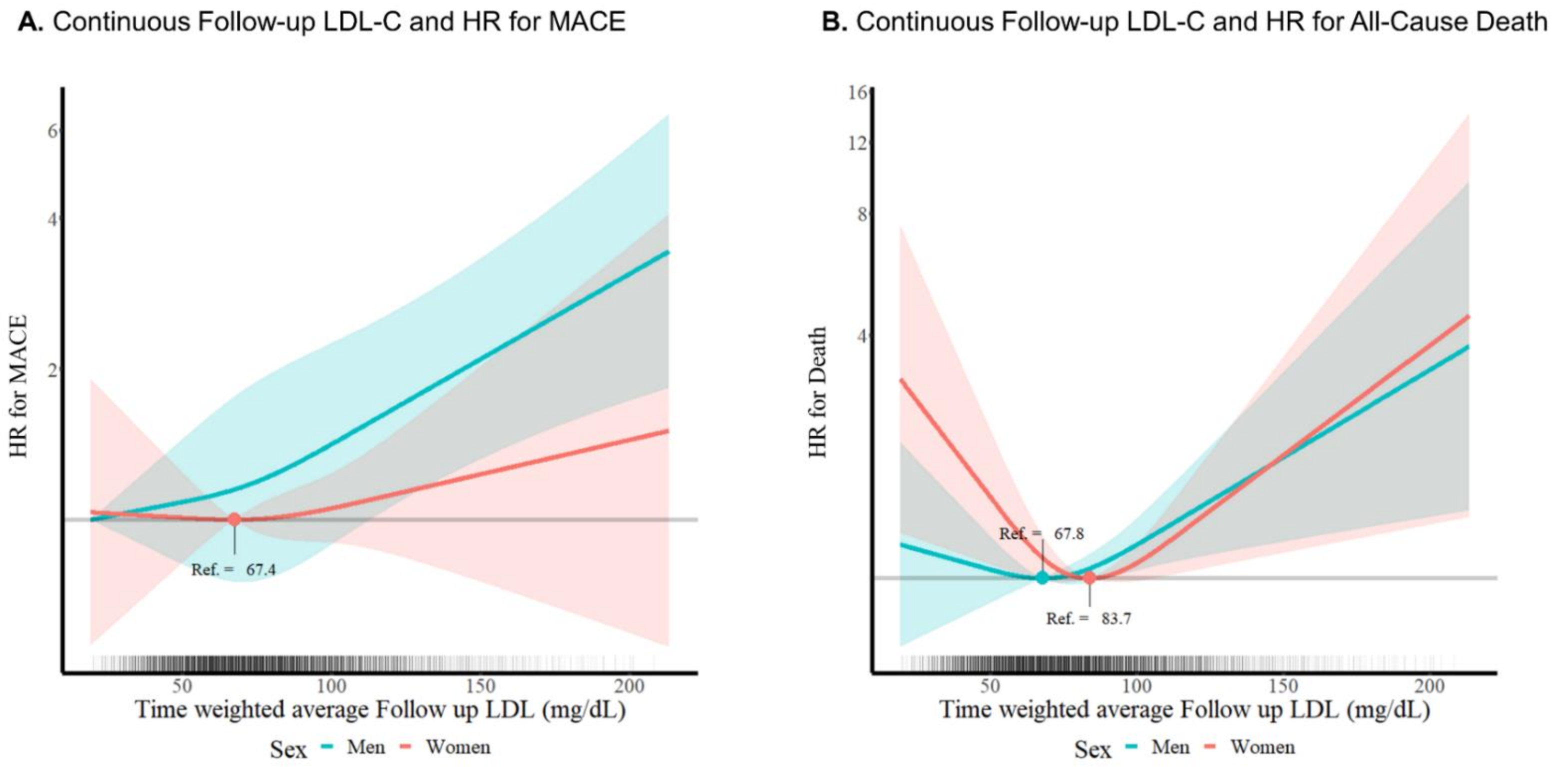

(A) Hazard ratios for major adverse cardiovascular events (MACE) at 5 years according to the continuous absolute follow-up LDL-C levels are shown. (B) Hazard ratios for all-cause death at 5 years according to the continuous absolute follow-up LDL-C levels are shown. Both were adjusted for age, sex, body mass index, hypertension, diabetes mellitus, current smoker status, family history of premature coronary artery disease, previous myocardial infarction, previous myocardial revascularization, ST-elevation myocardial infarction diagnosis, and statins medication.

Figure S4. Percentage low-density lipoprotein cholesterol (LDL-C) reduction rate and cardiovascular (CV) outcomes at 5 years stratified by sex. (Men, n = 4891; Women, n = 1357)

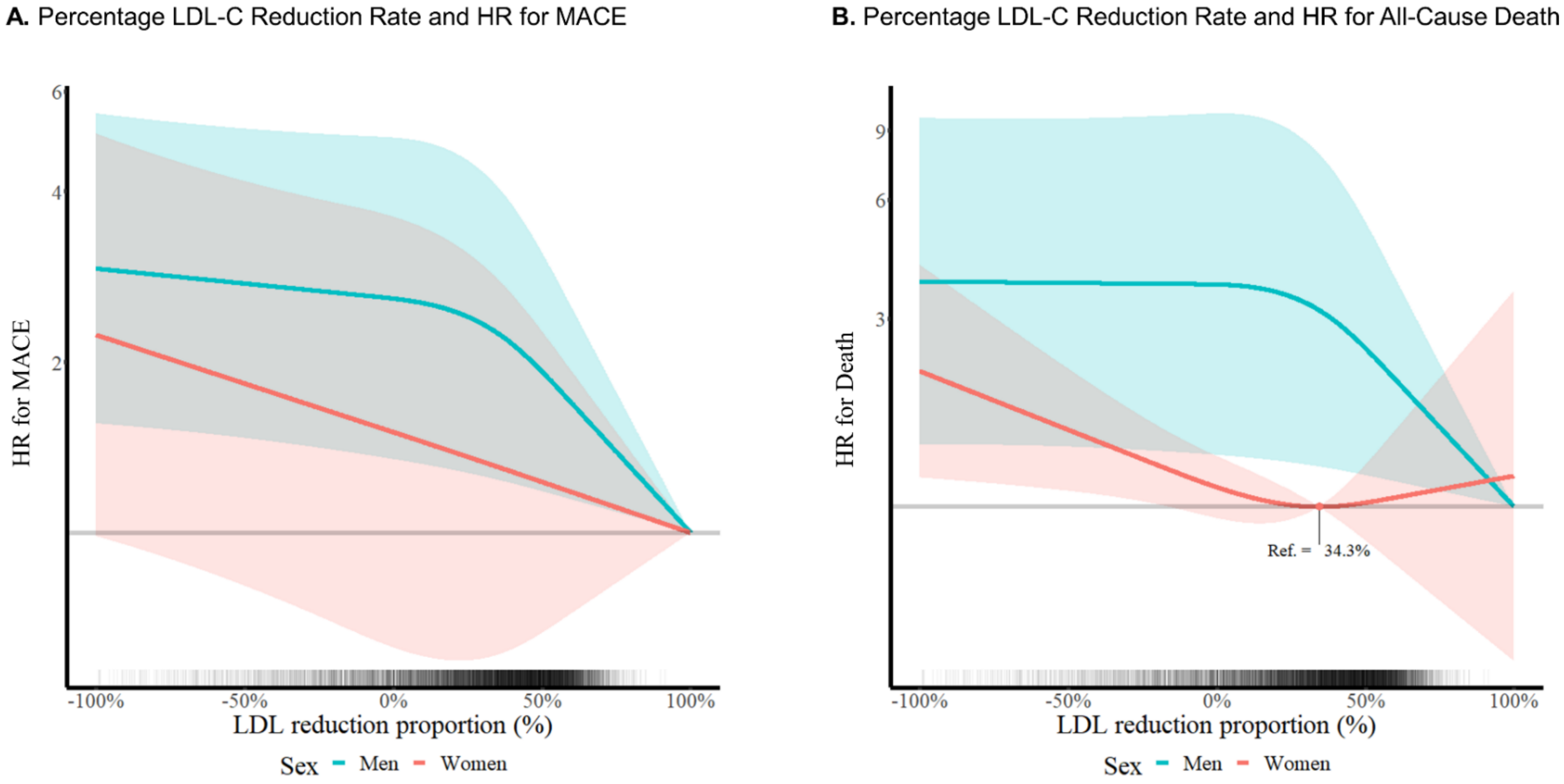

(A) Hazard ratios for major adverse cardiovascular events (MACE) at 5 years according to the percentage LDL-C reduction rate are shown. (B) Hazard ratios for all-cause death at 5 years according to the continuous percentage LDL-C reduction rate are shown. Both were adjusted for age, sex, body mass index, hypertension, diabetes mellitus, current smoker status, family history of premature coronary artery disease, previous myocardial infarction, previous myocardial revascularization, ST-elevation myocardial infarction diagnosis, and statins medication.
